# Surgery with versus without preoperative concurrent chemoradiotherapy for mid/low rectal cancer: an interim analysis of a prospective, randomized trial

**DOI:** 10.1186/s40880-015-0024-8

**Published:** 2015-06-10

**Authors:** Wen-Hua Fan, Fu-Long Wang, Zhen-Hai Lu, Zhi-Zhong Pan, Li-Ren Li, Yuan-Hong Gao, Gong Chen, Xiao-Jun Wu, Pei-Rong Ding, Zhi-Fan Zeng, De-Sen Wan

**Affiliations:** State Key Laboratory of Oncology in South China; Collaborative Innovation Center for Cancer Medicine, Sun Yat-sen University Cancer Center, Guangzhou, Guangdong 510060 P. R. China; Department of Colorectal Surgery, Sun Yat-sen University Cancer Center, Guangzhou, Guangdong 510060 P. R. China; Department of Radiation Oncology, Sun Yat-sen University Cancer Center, Guangzhou, Guangdong 510060 P. R. China

**Keywords:** Rectal cancer, Total mesorectal excision, Chemoradiotherapy, Oxaliplatin, Capecitabine

## Abstract

**Introduction:**

Multimodality therapy, including preoperative chemoradiotherapy (CRT) and total mesorectal excision (TME), has effectively reduced local recurrence rates of rectal cancer over the past decade. However, the benefits and risks of the addition of neoadjuvant CRT to surgery need to be evaluated. This study was to compare the efficacy of TME with versus without preoperative concurrent chemoradiotherapy (CCRT) involving XELOX regimen (oxaliplatin plus capecitabine) in Chinese patients with stages II and III mid/low rectal adenocarcinoma.

**Methods:**

We randomly assigned patients to the TME group (TME without preoperative CCRT) or CCRT + TME group (TME with preoperative CCRT). The primary endpoint was disease-free survival (DFS); the secondary endpoints were overall survival (OS), local and distant recurrence, tumor response to CRT, toxicity, sphincter preservation, and surgical complications. An interim analysis of the potential inferiority of DFS in the CCRT + TME group was planned when the first 180 patients had been followed up for at least 6 months.

**Results:**

A total of 94 patients in the TME group and 90 patients in the CCRT + TME group were able to be evaluated. The 3-year DFS and OS rates were 86.3 % and 91.5 % in the whole cohort, respectively. The 3-year DFS rates of the TME and CCRT + TME groups were 85.7% and 87.9 % (*P* = 0.766), respectively, and the 3-year OS rates were 90.7 % and 92.3 % (*P* = 0.855), respectively. The functional sphincter preservation rates of the TME and CCRT + TME groups were 71.3 % and 70.0 % (*P* = 0.849), respectively. In the TME group, 16 (17.0 %) patients were proven to have pTNM stage I disease after surgery. In the CCRT + TME group, 32 (35.6 %) patients achieved a pathologic complete response (pCR).

**Conclusions:**

Preliminary results indicated no significant differences in the DFS, OS, or functional sphincter preservation rates between the TME and CCRT + TME groups. However, preoperative CCRT with XELOX yielded a high pCR rate. Newer techniques are needed to improve the staging accuracy, and further investigation is warranted.

**Clinical trial registration number:**

Chi CTR-TRC-08000122

## Background

In China, colorectal cancer is the fifth most common cancer in male and the third most common cancer in female [1]. In 2014, 40,000 new cases of rectal cancer (23,380 male cases and 16,620 female cases) and 50,310 deaths were estimated in the United States [2]. Local recurrence and distant metastasis are major treatment failures of rectal cancer. In the last few decades, improvements in surgical techniques such as total mesorectal excision (TME) have reduced the local recurrence rates to <8 % [3–5]. In Western countries, the addition of (neo) adjuvant therapy has led to improvements in post-TME local control [6]. Based on current evidence, the gold standard for the treatment of stages II and III rectal cancer includes neoadjuvant chemoradiotherapy (CRT) before TME followed by postoperative chemotherapy [7, 8].

Although studies have confirmed that preoperative 5-fluorouracil (5-FU)-based CRT can reduce the local recurrence rate in colorectal cancer [6, 9], distant metastasis remains uncontrolled. The challenge is to integrate a new active agent to enhance the systemic control efficacy of neoadjuvant treatment. When combined with 5-FU, oxaliplatin has been shown to increase the tumor response in cases of metastatic colorectal cancer [10] and to be a potent radiosensitizing agent in preclinical studies [11, 12]. Furthermore, a recent meta-analysis suggests that the addition of oxaliplatin might improve the pathologic complete response (pCR) rate and reduce the perioperative metastatic rate [13].

However, the benefit of the addition of neoadjuvant CRT to surgery as compared with surgery without neoadjuvant CRT should be analyzed in terms of local control, toxicity, survival, and quality of life. The adverse effects of CRT, which include fecal incontinence, sexual, urinary, and bowel dysfunction, and secondary malignancy, can impair the quality of life of the patients and reduce life expectancy [14–16]. Furthermore, unnecessary neoadjuvant treatment gives great financial burden for patients. On the other hand, TME is a difficult surgery, and well-performed surgery has been shown to be a major short- and long-term prognostic factor of rectal cancer. Increased body mass index (BMI) is associated with a higher likelihood of local recurrence in patients with rectal cancer [17, 18]. Chinese patients always have a lower BMI compared with European patients, indicating better survival in the former group. Recent evidence suggests that the overtreatment of patients with CRT leads to unnecessary exposure to radiation and acute and long-term toxicity of radiotherapy, and it remains unclear whether TME without neoadjuvant CRT is adequate in treating the vast majority of Chinese patients. Therefore, in the present study, we compared the efficacy of TME with versus without preoperative concurrent chemoradiotherapy (CCRT) with capecitabine plus oxaliplatin (XELOX) in Chinese patients with stage II and III mid/low rectal adenocarcinoma.

## Materials and methods

### Study design

The present study was designed as a prospective, randomized phase II trial. The primary endpoint was disease-free survival (DFS); the secondary endpoints were overall survival (OS), local and distant recurrence, tumor response to CRT, toxicity, sphincter preservation, and surgical complications. We hypothesized that the 3-year DFS rate of patients who underwent either CCRT followed by TME (the CCRT + TME group) or TME without CCRT (the TME group) would be 80 %; a sample size of 252 patients per group achieved 80 % power to detect a noninferiority margin difference of −10 % between the group proportions. The CCRT + TME group proportion was assumed to be 70 % under the null hypothesis of inferiority. The power was computed for a case in which the actual study arm proportion was 80 %. The test statistic used was the 1-sided *z* test (unpooled). The significance level of the test was set at 0.025. An interim analysis was designed when the first group of 180 patients had completed all therapies and was followed up for at least 6 months.

### Patient selection

The enrollment criteria were as follows: pathologically confirmed rectal adenocarcinoma within 10 cm from the anal verge, the presence of clinical T3–T4 or node-positive resectable tumor, no extension of the malignant disease to the anal canal, and no evidence of distant metastasis. Tumor stage was determined according to the 2002 American Joint Committee on Cancer (AJCC) staging system. The staging workup included colonofiberscopy, endorectal ultrasonography (ERUS), chest computed tomography (CT), abdominopelvic CT, and/or abdominopelvic magnetic resonance imaging (MRI). Rigid sigmoidoscopy was also performed to determine the actual distance of the tumor from the anal verge. Further inclusion criteria were a Karnofsky Performance Scale score ≥70 points, age between 18 and 70 years, and adequate bone marrow function (hemoglobin level ≥100 g/L, white blood cell count ≥3.5 × 10^9^/L, absolute neutrophil count ≥1.5 × 10^9^/L, platelet count ≥100 × 10^9^/L), renal function (creatinine ≤1.5 × the upper limit of the normal range [ULN]), and hepatic function (aspartate aminotransferase/alanine aminotransferase [AST/ALT] ≤2.5 × ULN, alkaline phosphatase ≤2.5 × ULN).

The exclusion criteria included previously administered pelvic radiotherapy or chemotherapy, inflammatory bowel disease, malabsorption syndrome, a history of other cancers, cardiac arrhythmia, coronary heart disease, peripheral neuropathy, and psychiatric disorders or psychologic disabilities that might adversely affect treatment compliance. Pregnant or lactating women and women of childbearing potential who lacked effective contraception were also excluded.

### Ethics

The Sun Yat-sen University Cancer Center Institutional Review Board on Medical Ethics approved this study, and we performed the study in accordance with the Declaration of Helsinki. All patients provided written informed consent.

### Randomization and treatment

Patients were randomly allocated (1:1) by a computer-generated scheme, and their identities were concealed in sequentially numbered, opaque, sealed envelopes; the patients were then divided into the TME and CCRT + TME groups.

### Radiotherapy

Three-dimensional (3D) conformal radiotherapy was planned with the Pinnacle 8 treatment planning system (Philips, Amsterdam, Netherlands) using a 3-field irradiation technique with 8-MV X-rays. The gross tumor volume (GTV) was defined as all known gross lesions, including abnormally enlarged regional lymph nodes. The clinical target volume (CTV) included primary rectal tumor lesions, the two end portions of the rectum, perirectal tissues, and anterior sacral, iliac, obturator, and true pelvic internal iliac lymph drainage areas. In patients with T4 lesions or bladder-invading tumors, the CTV also included the external iliac lymph drainage area. The planned target volume (PTV) was defined as the CTV or GTV with 8-mm margin extension. Before 2011, a total dose of 46 Gy was delivered to the CTV in 23 fractions of 2 Gy each without a boost dose. From 2011 onwards, an addition of a 4-Gy boost dose that involved 2 fractions of 2 Gy each to the GTV increased the total dose to 50 Gy.

### Chemotherapy

Patients in the CCRT + TME group received 2 cycles of a modified XELOX regimen (oxaliplatin at 100 mg/m^2^ on Day 1 and capecitabine at 1,000 mg/m^2^ twice daily on Days 1–14 with an interval of 7 days) before TME,4 cycles of standard XELOX regimen (oxaliplatin at 130 mg/m^2^ on Day 1 and capecitabine at 1,000 mg/m^2^ twice daily on Days 1–14 with an interval of 7 days), and 2 cycles of capecitabine (1,000 mg/m^2^ twice daily on Days 1–14 with an interval of 7 days) after TME. In the TME group, patients with postoperative pathologic stages II–III disease were recommended to receive 6 cycles of standard XELOX regimen. All patients received standard antiemetic prophylaxis that consisted of 5-hydroxytryptamine receptor 3 (5-HT3R) antagonists and dexamethasone.

### Surgery

Patients underwent TME according to a standardized technique within 6–10 weeks after the completion of CRT. The surgeon made decisions regarding a covering stoma during the surgery. When the completeness of the TME was doubted, a frozen section of the mesorectal margin was subjected to intraoperative pathologic examination.

### Histopathologic assessment of the response to CRT

All patients underwent a diagnostic tumor biopsy before treatment. Sections with 4-mm thickness were obtained from a representative formalin-fixed, paraffin-embedded tumor tissue block. Pathologic evaluation of surgically resected specimens included tumor–node–metastasis (TNM) categorization, stage grouping, numbers of examined and involved lymph nodes, and tumor differentiation. A pathologic complete response (pCR) was defined as the complete disappearance of tumor cells. Primary tumor regression was semiquantitatively determined by the amount of viable tumor vs. the amount of fibrosis, which ranged from no evidence of treatment effect to complete response (CR) with no viable tumor identified, according to the Dworak regression grading system, as follows [19]: 0 (no regression), 1 (dominant tumor with fibrosis in 25 % of the tumor mass), 2 (dominant tumor with fibrosis in 26 %–50 % of the tumor mass), 3 (>50 % tumor regression), and 4 (complete regression).

### Toxicity evaluation and intervention

The patient’s medical history, clinical examination results, blood counts, and biochemistry results, including liver function, were monitored weekly. We used the National Cancer Institute Common Toxicity Criteria (NCI-CTC) version 3.0 to grade the toxicity. If an adverse effect > grade 2 (hematologic or gastrointestinal) was determined to be primarily chemotherapy-related, chemotherapy was discontinued until the toxicity resolved to grade 0–1. Capecitabine and oxaliplatin doses were adjusted for adverse events according to a previously described standard procedure [20]. If an adverse effect > grade 2 (hematologic or gastrointestinal) was determined to be primarily radiotherapy-related, radiotherapy was discontinued until the toxicity resolved to grade 0–1.

### Follow-up

The follow-up protocol included evaluations every 3 months for the first 2 years after the completion of all treatments and every 6 months thereafter. Evaluations at each visit included complete blood count, liver function test, carcinoembryonic antigen (CEA) and cancer antigen 19–9 (CA19-9) measurements, and physical examination. Chest, abdominal, pelvic CT, pelvic endoscopic ultrasonography, and/or MRI were conducted every 6 months during follow-up. Every follow-up was recorded in our database. The cutoff date for this trial was April 15, 2014.

### Statistical analyses

Survival curves were constructed using the Kaplan–Meier method and compared using the log-rank test. The pretreatment characteristics were compared using the Pearson chi-square test and the independent sample *t*-test. A Cox regression model was used for the multivariate analysis. All *P* values were 2-tailed and were considered significant when <0.05. Statistical analyses were performed with SPSS 13 for Windows (SPSS, Inc., Chicago, IL, USA).

## Results

### Clinicopathologic characteristics

Trial recruitment began on March 23, 2008, and ended on August 2, 2012, after we had enrolled 192 patients from a single institution. Of these 192 patients, 95 underwent surgery without preoperative CCRT (the TME group), and 97 underwent preoperative CCRT and surgery (the CCRT + TME group). Eight patients (4.2 %) were ineligible: in the TME group, distant metastasis was found in 1 patient during surgery; in the CCRT + TME group, distant metastasis was found in 3 patients during surgery, and 4 rejected surgery after preoperative CCRT. Forty-eight patients did not receive adjuvant chemotherapy because of refusal or unsuitability. The median age was 58 (range, 29–70) in the TME group and 56 (range, 28–70) in the CCRT + TME group (*P* = 0.219). Table 1 lists the baseline patient characteristics. The demographic and treatment characteristics of the two groups were comparable.

**Table 1:** Pretreatment characteristics of 184 patients with mid/low rectal cancer

Variable	TME group (cases [%])	CCRT + TME group (cases [%])	*χ* ^2^	*P*
Total	94	90		
Sex			1.199	0.273
Male	51 (54.3)	56 (62.2)		
Female	43 (45.7)	34 (37.8)		
Distance from the lower tumor margin to the anal verge			1.119	0.290
≤5 cm	47 (50.0)	52 (57.8)		
>5-10 cm	47 (50.0)	38 (42.2)		
T stage			6.833	0.033
cT2	8 (8.5)	2 (2.2)		
cT3	69 (73.4)	60 (66.7)		
cT4	17 (18.1)	28 (31.1)		
N stage			3.305	0.069
cN0	48 (51.1)	33 (36.7)		
cN+	46 (48.9)	57 (63.3)		
Clinical stage			2.752	0.097
II	48 (51.1)	33 (36.7)		
III	46 (48.9)	57 (63.3)		

TME, total mesorectal excision; CCRT, concurrent chemoradiotherapy

### Chemotherapy dose and compliance

Patients received a total of 1,007 capecitabine-based chemotherapy cycles. The CCRT + TME group received 2 cycles (total, 180 cycles) of capecitabine-based chemotherapy before TME and a median of 5 (range, 0–6; total, 396) cycles of capecitabine-based chemotherapy; the TME group received a median of 6 (range, 0–8; total, 431) cycles of capecitabine-based chemotherapy. All chemotherapy cycles were completed in both groups.

### Tumor response to CRT in the CCRT + TME group

The pCR rate in the CCRT + TME group was 35.6 % (32/90). Nodal status down-staging (cN+ to post-treatment pN0) was detected in 43 (75.4 %) of 57 patients, and the T category was down-staged in 68 (75.6 %) of 90 patients (Table 2). In all, 36 (40.0 %) patients exhibited grade 2 or 3 tumor regression.

**Table 2:** Pathologic stage of the 90 patients in the CCRT + TME group (cases)

Baseline stage	ypT0	ypT1	ypT2	ypT3	ypT4	Total	Baseline stage	ypN0	ypN1	ypN2	Total
T2	2	0	0	0	0	2	N-	27	6	0	33
T3	25	1	15	18	1	60	N+	43	12	2	57
T4	7	1	5	12	3	28	-	-	-	-	-
Total	34	2	20	30	4	90	Total	70	18	2	90

yp, pathologic stage after CCRT

### CCRT safety and dose intensity

All patients were able to be evaluated for toxicity. No treatment-related deaths occurred, and no patient developed grade 4 toxicity. Table 3 lists the adverse events that were observed during CCRT. Diarrhea was the most commonly observed event; 10 patients (11.1 %) developed grade 3 diarrhea. All grade 3 diarrhea events lasted less than 72 h because of adequate treatment with loperamide and oral fluid intake. No patient developed renal failure because of diarrhea. The hematologic toxicity was mild, and only 3 patients developed grade 3 leukocytopenia. No neutropenic fever was reported, and no patient received prophylactic or therapeutic granulocyte colony-stimulating factor during CCRT. Only 3 patients developed grade 1 anemia. Prophylactic recombinant human erythropoietin was not given, and no blood transfusions were required during CCRT. Grades 1–2 AST/ALT elevation, which was observed in 8 patients, might have been a consequence of the chemotherapy. Radiotherapy was administered, as prescribed, to all patients: 71 (78.9 %) received 46 Gy via 3D conformal radiotherapy, and 19 (21.1 %) received 50 Gy via intensity-modulated radiotherapy.

**Table 3:** Adverse events during CCRT for the 90 patients in the CCRT + TME group (cases)

Adverse event	NCI-CTC grade				
	0	1	2	3	4
Hematologic toxicity					
Anemia	87	3	0	0	0
Thrombocytopenia	84	5	1	0	0
Leukocytopenia	74	5	8	3	0
Non-hematologic toxicity					
Diarrhea	50	16	14	10	0
Nausea/vomiting	62	18	10	0	0
Stomatitis	80	10	0	0	0
Abdominal pain	78	8	4	0	0
Proctitis	58	16	16	0	0
Metabolic/laboratory abnormality					
AST/ALT elevation	82	6	2	0	0
Bilirubin elevation	86	3	1	0	0
Creatinine elevation	89	1	0	0	0
Radiation-related toxicity					
Hand-foot skin reaction	73	13	4	0	0
Dermatitis	66	16	8	0	0

AST, aspirate aminotransferase; ALT, alanine aminotransferase; NCI-CTC, National Cancer Institute Common Toxicity Criteria

### Resectability and sphincter preservation

R0 resection was achieved in all patients. Furthermore, 16 (17.0 %) patients in the TME group were shown to have pTNM stage I disease after surgery. The staging accuracy in the TME group was 62.8 % (59/94) for T category (16 [17.0 %] up-staged and 19 [20.2 %] down-staged) and 41.5 % (39/94) for N category (28 [29.8 %] up-staged and 27 [28.7 %] down-staged). The overall functional sphincter preservation rate was 70.7 % (130/184). The functional sphincter preservation rates were 71.3 % (67/94) in the TME group and 70 % (63/90) in the CCRT + TME group (*P* = 0.849). The proportions of patients with tumors within 5 cm from the anal verge were similar in the CCRT + TME and TME groups (57.8 % vs. 50.0 %, *P* = 0.290).

### Surgical complications

Table 4 lists the surgical parameters and complications. No postoperative deaths occurred in either group. The rates of anastomotic leakage were 8.5 % in the TME group and 2.2 % in the CCRT + TME group (*P* = 0.101). The rates of genitourinary symptoms, obstruction, colostomy, and wound infections were also similar in both groups.

**Table 4:** Surgical parameters and tumor regression after treatment in the TME and CCRT + TME groups

Variable	TME group	CCRT + TME group	*χ* ^2^	*P*
Total (cases)	94	90		
Sphincter preservation (cases [%])			0.036	0.849
Yes	67 (71.3)	63 (70.0)		
No	27 (28.7)	27 (30.0)		
Time of surgery (hours)			−1.106	0.269
Median	3	3		
Range	2-6	2-10		
Blood loss during surgery (mL)			−0.387	0.698
Median	100	100		
Range	20-1,000	50-1,000		
Hospital stay (days)			−0.540	0.589
Median	9	9		
Range	6-33	6-98		
Complications (cases [%])				
Urinary symptoms	0	2 (2.2)		0.238
Anastomotic leakage	8 (8.5)	2 (2.2)		0.101
Obstruction	1 (1.1)	1 (1.1)		1.000
Temporary colostomy	0	3 (3.3)		0.115
Wound infection	4 (4.3)	4 (4.4)		1.000
Tumor regression grade^a^ (cases [%])				
4	NA	34 (37.8)		
3	NA	23 (25.6)		
2	NA	13 (14.4)		
1	NA	20 (22.2)		
pTNM stage (cases [%])			3.202	0.202
T1-2N0M0	16 (17.0)	17 (18.9)		
T3-4N0M0	39 (41.5)	21 (23.3)		
T1-4 N1-2 M0	39 (41.5)	20 (22.2)		
pCR (cases [%])	NA	32 (35.6)		

pCR, pathologic complete response (ypT0N0); NA, not applicable. Other abbreviations as in Table 1. ^a^The Dworak regression grading system was used for tumor regression grading

### Tumor control and survival

**Fig. 1:**
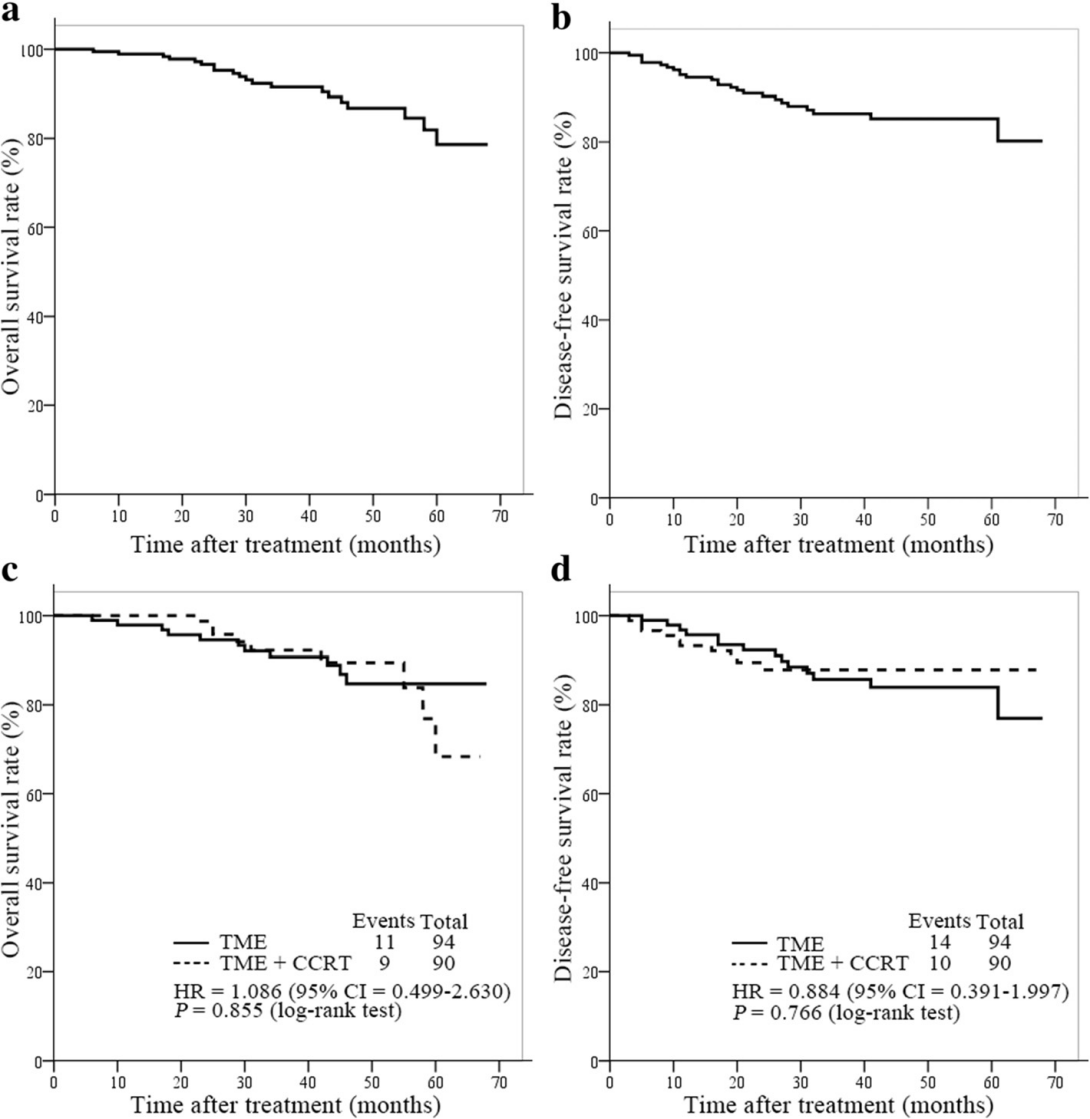
Kaplan-Meier survival curves of the patients with mid/low rectal cancer in the TME and CCRT + TME groups. TME, total mesorectal excision; CCRT, concurrent chemoradiotherapy; HR, hazard ratio; CI, confidence interval. **a**, the overall survival (OS) curve of the whole cohort. **b**, the disease-free survival (DFS) curve of the whole cohort. **c**, the OS curves of the TME and CCRT + TME groups. **d**, the DFS curves of the TME and CCRT + TME groups.

**Fig. 2:**
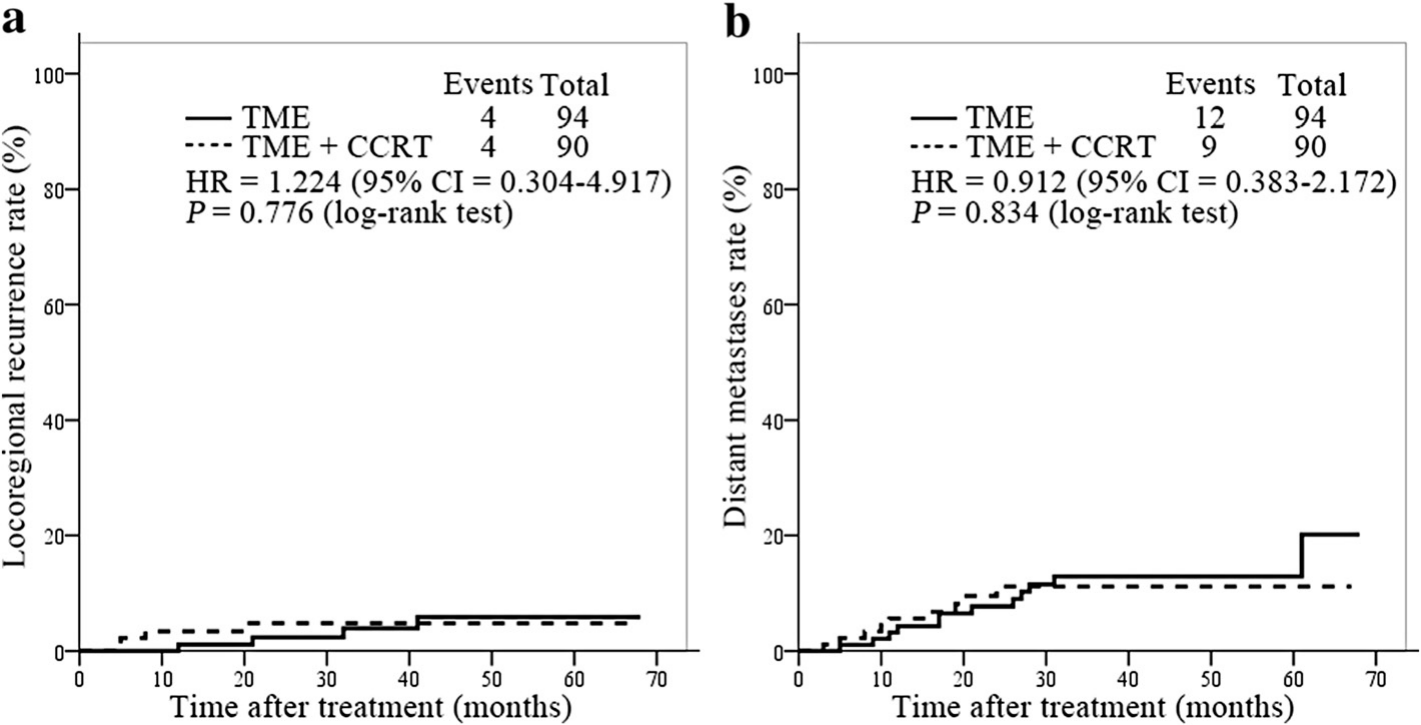
Kaplan-Meier curves of local recurrence and distant metastasis in the TME and CCRT + TME groups. **a**, local recurrence rates were similar in the TME and CCRT + TME groups. **b**, distant metastasis rates were similar in the TME and CCRT + TME groups.

At the time of the analysis (April 2014), the median follow-up period was 38 (range, 4–68) months for all patients, 43 (range, 4–67) months for the CCRT + TME group, and 43.5 (range, 6–68) months for the TME group (*P* = 0.825). The 3-year DFS and OS rates were 86.3 % and 91.5 %, respectively, in the whole cohort. No significant differences in DFS and OS were found between the two groups (Table 5). Fig. 1 depicts the survival curves of patients in the TME and CCRT + TME groups. In the TME group, 2 patients developed local recurrence, 10 developed distant metastasis, and 2 developed both local recurrence and distant metastasis; in the CCRT + TME group, 1 patient developed local recurrence, 6 developed distant metastasis, and 3 developed both local recurrence and distant metastasis (Fig. 2, Table 5).

**Table 5:** Tumor control and survival of the patients in the TME and CCRT + TME groups

Group	3-year rate (%)		Local recurrence (cases [%])	Distant metastasis (cases [%])
	DFS	OS		
TME	85.7	90.7	4 (4.3)	12 (12.8)
CCRT + TME	87.9	92.3	4 (4.4)	9 (10.0)
*P*	0.766	0.855	0.776	0.834

## Discussion

This trial failed to show differences in DFS, OS, and local recurrence between the TME and CCRT + TME groups. The cumulative incidences of local recurrence were only 4.3 % in the TME group and 4.4 % in the CCRT + TME group. The 3-year DFS rates were 85.7 % in the TME group and 87.9 % in the CCRT + TME group (*P* = 0.766); the 3-year OS rates were 90.7 % and 92.3 % (*P* = 0.855), respectively. The sphincter preservation rates in our study (70.7 %) were similar to those reported in previous phase III studies (60 %–75 %) [21, 22]. Moreover, we detected nodal status and T category down-staging in 75.4 % and 75.6 % of patients, respectively. Several possible reasons might explain the similar results obtained in both groups.

Incorrect preoperative staging may affect treatment decision, even leading to overtreatment. In our study, most patients (83.2 %) underwent abdominopelvic CT for staging. The staging accuracy in the TME group was 62.8 % for T stage (17.0 % up-staged and 20.2% down-staged) and 41.5 % for N stage (29.8 % up-staged and 28.7 % down-staged). Sixteen (17.0%) patients in the TME group were proven to have pTNM stage I disease after surgery. We believe that the CCRT + TME group would contain a similar percentage of patients with pTNM stage I disease and that these patients would not benefit from preoperative CCRT. Therefore, preoperative staging accuracy is considered to be among the factors that lead to similarities between the groups.

Currently, new techniques such as MRI or positron emission tomography (PET) are used to improve the accuracy of lymph node staging [23, 24]. High-resolution MRI can more accurately and reproducibly predict the tumor stages of rectal cancer [25]. Fluorodeoxyglucose (FDG) PET/CT appears to be less accurate than MRI for lymph node staging which is due to its inability to detect lymph node micrometastases (<5 mm) and the “blooming effect” of primary hot lesions that overshadow the nearest lymph nodes [26]. Nevertheless, several studies have reported a higher specificity of FDG PET/CT compared with MRI (83 %–85 % vs. 67 %) in terms of nodal staging, suggesting that in cases of rectal cancer, a glucose-avid lymph node is most likely positive [27, 28]. Based on these findings, PET/CT provides additional benefits for the discrimination of metastatic lymph nodes in patients with rectal cancer. Additionally, combined MRI and PET (fusion imaging) will be expected to increase the accuracy of nodal staging predictions.

Another possible reason for the similarities between the two groups in our study might be the BMI of these Chinese patients. TME is a difficult surgery, and the quality of the surgery may be influenced by several factors such as a narrow pelvic diameter on MRI and pathologic BMI (either above or below the normal range). A high BMI has been associated with technical difficulty in the surgical procedure and increased perioperative complications, which may result in an increased risk of local recurrence in patients with rectal cancer [17, 18]. Chinese patients might therefore benefit from a lower BMI as compared with patients from Western countries. In our study, all patients had R0 resection compared with 96.8 % of European and Canadian patients [29]. Consequently, neoadjuvant CRT may not be as vital for some Chinese patients. Furthermore, the small sample sizes and the relatively short follow-up periods of previous trials might have limited the ability to detect moderate but clinically important differences.

The most likely explanation for the similarities between the two groups in our study is that oxaliplatin may not be a clinically effective radiation sensitizer. Based on its successful use in colorectal cancer, oxaliplatin was used in combination with radiation, and existing preclinical data demonstrated the synergistic effects of oxaliplatin and radiation [30–32]. Furthermore, early clinical trials reported promising activity when oxaliplatin was added to 5-FU-based CRT in patients with stages II–III rectal cancer [11, 12, 19, 33]. However, data from phase III trials suggest that oxaliplatin induces toxicity without conferring a clinical benefit and therefore should not be included in standard preoperative CRT [22, 34]. Intriguingly, the pCR rate of the CCRT + TME group in our study was higher than the rates reported by several phase III clinical trials that combined radiation and the XELOX regimen (35.6 % vs. 16 %–21 %) [35]. We also found that only 9 (10.0 %) patients in the CCRT + TME group developed distant metastasis compared with 12 (12.8 %) in the TME group. The pCR rate in our study (35.6 %) was also much higher than those in phase II studies that used capecitabine and radiotherapy as neoadjuvant treatments for locally advanced rectal cancer, in which the reported pCR rates only ranged from 7 % to 24 % [36]. The modified XELOX regimen used in our trial apparently led to a higher pCR rate compared with regimens that included weekly oxaliplatin.

Our trial suggests that CRT with capecitabine and oxaliplatin is well tolerated in patients with locally advanced rectal cancer. No postoperative deaths occurred in either group. The rates of urinary symptoms, obstruction, colostomy, and wound infections were similar between the groups, as were the duration of surgery, blood loss during surgery, and the duration of hospital stay. The potential adverse effects of various radiotherapy and chemotherapy combinations are currently being investigated in patients with locally advanced rectal cancer. In other countries, phase III clinical trials of neoadjuvant 5-FU and oxaliplatin combined with radiation for patients with rectal cancer have shown that the most frequently reported toxicity was grade 3/4 diarrhea, with incidence ranging from 12 % to 15 % [23, 35]. This is in accordance with the results of our study, where grade 3 diarrhea was the main toxicity, and we observed no increase in postoperative morbidity. Hematologic toxicity was mild. Therefore, the addition of oxaliplatin to a preoperative capecitabine-based radiotherapy regimen is safe and does not require dose reductions of the treatment components.

Interestingly, recent studies have shown that for selected patients with clinical stage II/III rectal cancer, neoadjuvant chemotherapy without radiotherapy might also be effective [37, 38]. However, it shall be noted that selective treatment strategies for these patients significantly rely on the ability of the imaging techniques to allow for an accurate identification of the stage of rectal tumors and the high-risk features at baseline. Additionally, the actual applicability of these results [37, 38] is limited by the sample size, the exclusion of most patients with “ugly tumors” (T4 tumors with overgrowth to the prostate, seminal vesicles, base of urinary bladder, pelvic side walls or floor, and sacrum; positive lateral lymph nodes; and positive circumferential resection margin [CRM]) [39], the lack of a control group treated with preoperative radiotherapy, established risk-adapted criteria for patient selection, and sufficient long-term follow-up.

Several limitations of the present study should be mentioned. We have presented only an interim analysis of the trial. The current sample size did not meet the designated requirements, and longer follow-up is ongoing to determine the definite role of the CCRT + TME strategy on local control and survival. In addition, the staging accuracy in the TME group was only 62.8 % for T stage and 41.5 % for N stage. New techniques such as MRI, ERUS, and even PET/CT are needed to improve the staging accuracy. Therefore, our results should be interpreted with caution.

## Conclusions

Preliminary results of the trial failed to show differences in DFS, OS, and sphincter preservation rates between the TME and CCRT + TME groups in Chinese patients. TME without preoperative CCRT may be adequate in treating the Chinese patients with stages II and III mid/low rectal adenocarcinoma, although longer follow-up and further research is needed to verify this finding. Adding oxaliplatin to fluorouracil-based preoperative chemoradiotherapy significantly increases short-term efficacy and can be safely combined with capecitabine plus radiotherapy in Chinese patients with rectal cancer. The long-term benefits are still under observation.
